# Retrospective analysis of pediatric patients with multiple rare-earth magnets ingestion: a single-center experience from China

**DOI:** 10.1186/s12887-021-02642-y

**Published:** 2021-04-17

**Authors:** Yucan Zheng, Zhihua Zhang, Kunlong Yan, Hongmei Guo, Mei Li, Min Lian, Zhifeng Liu

**Affiliations:** Department of Gastroenterology, Children’s Hospital of Nanjing Medical University, Nanjing, 210008 China

**Keywords:** Children, Foreign body, Multiple magnets ingestion, Perforation

## Abstract

**Background:**

The aim of this study was to characterize patients who ingested multiple rare-earth magnets, reveal the harm of rare-earth magnet foreign bodies in the digestive tract, and develop a clinical management algorithm.

**Methods:**

This was a retrospective review of patients with rare-earth magnet foreign bodies in the digestive tract admitted to a university-affiliated pediatric medical center in China, between January 2016 and December 2019; the subset of medical data evaluated included clinical symptoms, signs, treatments and outcomes.

**Results:**

A total of 51 cases were included in this study, including 36(70.6%) males and 15(29.4%) females. The magnets were passed naturally in 24(47.1%) patients and removed by intervention in 27(52.9%) patients, including 5(9.8%) cases by endoscopy and 22(43.1%) cases by surgery. Twenty-two (43.1%)cases had gastrointestinal obstruction, perforation, and fistula. Compared with the non-surgical group, the time of the surgical group from ingestion to arriving at the hospital was longer([80(5–336) vs 26(2–216)]hours, *p* < 0.001) while there was no significant difference in the mean age or the number of magnets swallowed.

**Conclusions:**

Magnets are attractive to children, but lead to catastrophic consequences including gastrointestinal obstruction, perforation, and surgical interventions when ingested multiple magnets. Endoscopic resection should be urgently performed in the presence of multiple magnets as early as possible within 24 h, even in asymptomatic patients.

## Background

Foreign body ingestion is quite common in pediatric clinical practice; however, the ingestion of multiple magnets is rare. Most foreign bodies without sharp edges, with moderate size and with corrosion resistance, such as toy accessories, coins, and rings, can be excreted from the digestive tract themselves. However, 1% of cases require surgical treatment for foreign body retention or associated complications [1]. Among the many different foreign bodies swallowed by children, the ingestion of multiple magnets is a unique situation. The ingestion of multiple magnets, especially when swallowed individually, may cause serious complications such as gastrointestinal perforation, internal fistula, intestinal obstruction and even death [2].

Recently, a new kind of rare-earth magnetic toy, named buckyballs, has become popular among children and adolescents. Multiple rare-earth magnets of different colors can be combined into different shapes, which are attractive to kids. In contrast to traditional magnets, buckyballs are spherical strong magnets made of neodymium iron boron (NdFeB)-a kind of permanent magnetic material, whose magnetic force is as high as 4000 G. Generally, a small buckyball with a diameter of five millimeters can hold two coins steadily. For the past 10 years, a small number of cases of intestinal perforation caused by the ingestion of strong magnets have been reported [2, 3]. We analyzed the clinical features of pediatric patients treated for the ingestion of multiple magnets at a single pediatric center, to emphasize the hazard of multiple rare-earth magnet ingestion and provide a reference for clinical practice.

## Methods

**Fig. 1:**
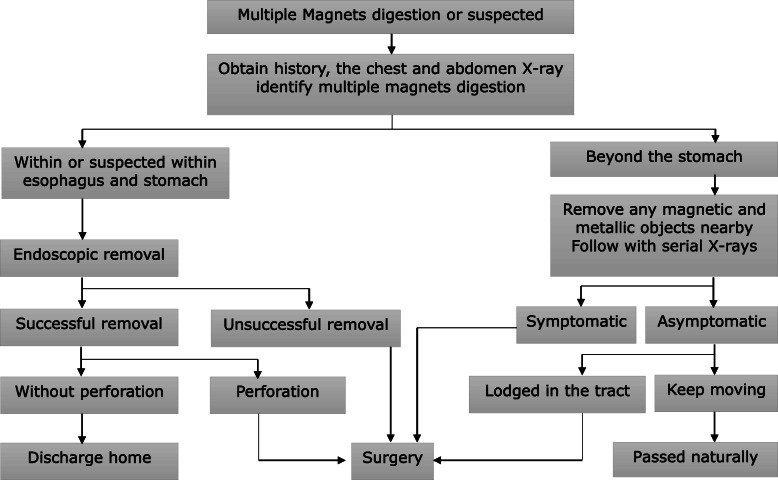
Algorithm of multiple rare-earth magnets ingestion management in children. †If a single magnet or multiple magnets ingestion cannot be defined then consider as multiple magnets. †Single magnet and metallic object consider as multiple magnets

We retrospectively reviewed the case records of all patients less than 18 years old who had ingested multiple rare-earth magnets ingestion and were treated at a university-affiliated pediatric medical center in China, from January 2015 to December 2019. The diagnoses of these patients were based on their history and X-ray results. Those cases with single magnet ingestions were excluded. We managed all the patents according to the algorithm shown in Fig. 1. Then we collected all clinical data from the included patients, including demographics, chief complaints, symptoms, physical signs, imaging data, endoscopy findings, surgical procedures, outcomes, and follow-up information.

Descriptive count data with normal distribution are represented by means±standard and the differences between two groups were evaluated by independent sample t test. While, data with non-normal distribution are represented by means (minimum-maximum), and the differences were evaluated by Mann-Whitney U test. The frequencies are reported as percentages. The difference of gender distribution between two groups was evaluated by chi-square test. All reported *p* values are two-tailed, and statistical significance was set at *p* < 0.05. Statistical analysis was performed using IBM SPSS Statistics 20(IBM Corp., Armonk, NY).

## Results

### General data

**Fig. 2:**
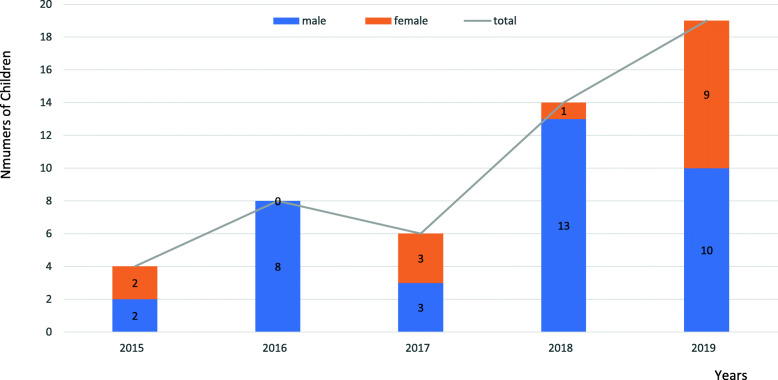
Numbers of children swallowed multiple magnets each year from 2015 to 2019

A total of 51 cases were included in this study. The mean age of these cases was 5.3 years. The proportion of male patients was 70.6%(36/51), which was higher than that of female patients. From 2015 to 2019, the number of cases increased (Fig. 2).

### Clinical manifestations

**Fig. 3:**
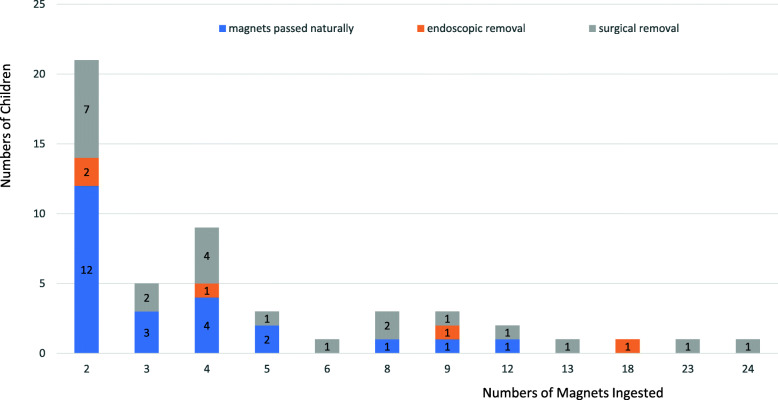
Numbers of magnets children swallowed

The numbers of magnets ingested ranged from two to twenty-four. The ingestion of two magnets accounted for the most cases (21/51, 41.2%)(Fig. 3). Among the 51 cases, the specific time of ingestion of was unknown for six children. The average visit time of the other 45 children was 46.8 h after their ingestion, ranging from 2 h to 14 days. Only 19 cases (37.3%) were symptomatic, including 15 cases of abdominal pain, 12 cases of vomiting, two cases of fever and one case of chest pain. Nine patients had positive signs of abdominal tenderness, and four of them had peritoneal irritation signs (Table 1).

**Table 1:** Clinical features and treatments

Symptoms, No. (%)	
No	32 (62.7%)
Yes	19 (37.3%)
Abdominal pain	15 (29.4%)
Nausea and vomiting	12 (23.5%)
Fever	2 (3.9%)
Chest pain	1 (2.0%)
Signs, No. (%)	
No	42 (82.4%)
Yes	9 (17.6%)
Abdominal tenderness only	9 (17.6%)
Peritoneal irritation signs	4 (7.8%)
Radiograph tests, No. (%)	
X-rays	51 (100%)
Foreign bodies only	49 (96.1%)
Bowel obstruction	2 (3.9%)
Clinical findings, No. (%)	
No adverse effects	26 (51.0%)
Mucosal injury	2 (3.9%)
Ulcer	1 (2.0%)
Magnets lodged in gastrointestinal tract	2 (3.9%)
Fistula	1 (2.0%)
Perforation	3 (5.9%)
Multiple perforations, adhesions and necrosis	16 (31.4%)
Treatments, No. (%)	
No intervention	18 (35.3%)
Endoscopy	10 (19.6%)
Surgery	16 (31.4%)
Endoscopy+ surgery	7 (13.7%)

### Clinical management

All 51 children had positive foreign body shadows on abdominal plain radiographs, of which two patients showed intestinal obstruction. Six cases also had CT examinations, which showed three cases of intestinal obstruction, one case of intestinal perforation, and two cases of gastrointestinal foreign bodies only.

According to the radiographs, 17 cases’ magnets were possibly still located in the upper digestive tract, so gastroduodenoscopies were performed. By gastroduodenoscopy, the magnets of five cases were removed, of which two cases had no complications, one case had slight mucosal injury and two cases had perforation. In one of these two cases, the location of the perforation was the duodenum, and the patient was subsequently transferred to surgery, underwent duodenal perforation repairment and jejunal nutrition tube placement. After treated by anti-infection and enteral nutrition for 14 days, the patient recovered and was discharged home. The perforation of the other case was in the lower esophagus. After 10 days of local drainage and enteral nutrition through a nasojejunal tube, the perforation healed. In six cases, the magnets had passed through the pylorus and entered the lower digestive tract, and the gastric mucosa had mild injury in one of these six cases. In the other six cases, the magnets were still in the upper digestive tract, but they were embedded in the mucosa and could not be removed by gastroduodenoscopy. Therefore, these six cases were transferred to surgery.

In this study, a total of 23 children underwent surgery, including 21 cases of laparotomy and two cases of laparoscopy combined with laparotomy. After the operation, four patients stayed in the ICU for monitoring for 2 or 3 days. Twenty-four patients fasted for an average of 7.5 days, ranging from 2-10 days. Twenty-two patients had gastrointestinal decompression for an average of 6.8 days, ranging from 2-9 days. Sixteen patients had abdominal drainage for an average of 7.8 days, ranging from 5-9 days. Eleven patients had fever after surgery for an average of 2.7 days, ranging from 1-6 days. Twenty-two patients used antibiotics for an average of 11.8 days, ranging from eight to 19 days.

The patients were divided into two groups according to whether they underwent surgery. Compared with the non-surgical group, the surgical group had more male cases; the time from ingestion to arriving at the hospital, fasting time, durations of antibiotics and the length of hospital stay were longer; the peripheral white blood cell count (WBC) and C-reactive protein were higher. However, there was no significant difference in the mean age or the number of magnets swallowed (Table 2).

**Table 2:** Difference between surgical and non-surgical group

	Surgery(*n* = 23)	No surgery(*n* = 28)	*P* value
Age, years	4.6 (1.7–9.3)	5.9 (0.3–10.9)	0.135
Gender, No (%)			0.004
Male	21 (91)	15 (54)	
Female	2 (9)	13 (46)	
The number of magnets	6 (2–24)	4 (2–18)	0.241
Visit time after ingestions, hours	80 (5–336)	26 (2–216)	< 0.001
Fasting time, days	0.3 (0–9)	7.4 (2–10)	< 0.001
Durations of antibiotics, days	0.3 (0–9)	11.9 (8–19)	< 0.001
Length of hospital stay, days	2.0 (0–11)	12.6 (8–19)	< 0.001
WBC, *10^9/L	7.2 ± 1.9	10.2 ± 3.7	0.001
CRP, mg/L	4 (4–4)	7.8 (4–30)	0.001

### Clinical findings and outcomes

Among the 51 cases, 26 of them had no visceral injury while 25 of them had gastrointestinal injuries, and the clinical features are shown in Table 1. In the most serious case, there were 11 intestinal perforations, three segments of intestinal necrosis, and extensive intestinal adhesion. In the 51 cases, the magnets passed naturally for 47.1% (24/51), were removed by endoscopies for 9.8% (5/51) and were removed by surgeries for 43.1% (22/51). Thirty-nine patients were hospitalized, the average length of hospital stay was 8.8 days, ranging from 1-9 days. The 23 patients undergone surgery were followed up for 6 months, and no postoperative complications occurred. No deaths occurred in these cases. We followed all patients for at least 6 months. There were no long-term complications, such as obstruction or infection.

## Discussion

Rare-earth magnets are not the most common foreign body that children swallow, but they require special attention because of the hazard they present. In the past 10 years, we have seen several reports of serious complications caused by multiple magnets ingestion from many countries [3–5]. Therefore, the USA government has forced toy companies to recall certain magnetic toys. Accordingly, in some areas of the USA, the incidence of multiple magnets ingestion has decreased [6]. In China, although there are warning labels on the toy packages, a recent multicenter investigation shown that the incidence of multiple magnets ingestions is increasing [7]. As our data show, the number of patients in 2019 was approximately four times higher than that in 2015. Previous studies have shown that 80–90% of foreign bodies pass spontaneously, and 1–5% of cases require surgical intervention [1, 8]. However, in our study, the surgical intervention rate was as high as 45.1%. It’s similar to that reported in another study of multiple magnets ingestion [3].

The magnetic force of the rare-earth magnet is 30–50 times stronger than that of ordinary magnet and each rare-earth magnet can attract 640 times its own weight. After multiple rare-earth magnets were swallowed, they attracted each other tightly, resulting in local compression of the intestinal wall, ischemia, necrosis, perforation, internal fistula, peritonitis, intestinal obstruction and even death [9]. The rare-earth magnets in toys that children swallow are generally small, and the perforation caused by their ingestion is also small. In some cases, because of the wrapping of the omentum after perforation or the formation of an internal fistula directly, the clinical symptoms are mild and the imaging findings are not typical. In this study, 20 cases had perforation, fistula, and obstruction, but five of them had mild or no symptoms and only four cases had typical changes on the radiographs, which is similar to several other reports [4, 10, 11]. Some children cannot provide the exact time of swallowing the magnets, which may delay the diagnosis and treatment. It is one of the causes of serious complications. In this study, a 3-year-old boy had no symptoms and was diagnosed 14 days after he swallowed the magnets when a small intestine perforation had already formed.

For cases with an uncertain number of magnets swallowed, it is necessary to treat them as if multiple magnets have been swallowed; multi-position photography may help to determine the number of magnets. For patients reporting that a single magnet swallowed, follow-up observation is also necessary. If the magnet position is fixed, the necessary intervention should be performed [9].

For the treatment of magnetic foreign bodies in the digestive tract of children, different guidelines have different recommendations. The 2017ESGE/ESPGHAN guidelines recommends urgent (< 24 h) removal of all magnets within endoscopic reach. For those beyond endoscopic reach, the guideline advises that close observation and surgical consultation [12]. The NASPGHAN guideline 2015 recommends different managements approaches based on whether the case is a single magnet ingestion or multiple magnets ingestion. For a single magnet ingestion, this guideline recommends removal if possible or confirmation of passage with serial X-rays. For multiple magnets ingestions within the stomach or esophagus, this guideline recommends removal by endoscopy if the time is< 12 h from ingestion. In cases > 12 h since ingestion, this guideline recommends consulting pediatric surgery prior to endoscopic removal. For multiple magnets beyond the stomach, the patient could be treated by enteroscopy or colonoscopy for removal if asymptomatic or followed with serial X-ray to check for progression [1]. However, if symptomatic, the patient should be referred to pediatric surgery [1]. In this study, patients were managed using the following algorithm (Fig. 1) which was based on the guidelines and the actual experiences at our center. For patients with perforation and fistula found after endoscopic removal, the general treatment is to switch to surgery [13]. However, one patient in this study had a fistula from the lower esophagus to the cardia. After removal, the fistula contracted, and the wound was small. Therefore, we did not perform surgery but gave gastric tube decompression and stopped his oral diet. No symptoms such as pneumoperitoneum or abdominal pain occurred. After 10 days, the gastroscope was reexamined and the wound had healed well. Then, the patient’s oral diet was restored, and the patient was discharged smoothly. Another case report shows that patients with mild symptoms of fistula did not undergo surgery and recovered smoothly after removal of the magnets by endoscopy [14].

In our study, surgical procedures performed in four situations: the magnets were in the upper gastrointestinal tract but cannot be remove by endoscopy; the magnets were removed by endoscopy but gastrointestinal perforation was found after removal; the magnets were far from the upper gastrointestinal tract but the patients had gastrointestinal symptoms during the followed period; the magnets were lodged in the tract. These patients in surgical group had a delayed visit time after ingestions and suffered from a longer duration for fasting, antibiotics use, and hospital stay. Therefore, to reduce the rate of surgery we appeal to patients for early medical consulting in case of magnets ingestions, and to doctors for conducting endoscopy to suspected patients as early as possible within 24 h. The WBC and CRP of the patients in surgical group were higher than that in non-surgical group, which suggests that if the patients have increased WBC and CRP, the doctors should alert that the patients may under a situation needs surgery.

Our study has several limitations. It was a retrospective chart review from a single hospital, and it was limited by the quality of documentation and the number of cases. In our study, we found the proportion of males in the surgical group was higher compared with the non-surgical group, which we cannot explain the reason. It may be limited by the size of the samples. Moreover, some of the patients were followed for only 6 months. It may be possible that some of the results cannot be sustained over a longer period.

## Conclusions

Multiple rare-earth magnets can cause serious gastrointestinal injury and have a high risk of requiring intervention for removal. Primary prevention is very important. Visible warnings and regular information over the package should be advertised to inform children and their parents involved in the use of these multiple rare-earth magnets. For parents and physicians, it is necessary to be aware of the dangers associated with the ingestion of multiple rare-earth magnets. If such ingestions are suspected, medical attention should be received promptly. Besides, endoscopic resection should be urgently performed in the presence of multiple magnets as early as possible within 24 h, even in asymptomatic patients.

## Data Availability

The datasets are available from the corresponding author on reasonable request.

## Abbreviations

NdFeB: Neodymium iron boron
ICU: Intensive care unit
WBC: White blood cell
CRP: C-reactive protein
ESGE/ESPGHAN: European Society of Gastrointestinal Endoscopy/European Society of Pediatric Gastroenterology, Hepatology and Nutrition
NASPGHAN: North American Society of Pediatric Gastroenterology, Hepatology and Nutrition
